# Case Report: Challenging Otologic Surgery in Patients With 22q11.2 Deletion Syndrome

**DOI:** 10.3389/fsurg.2020.00053

**Published:** 2020-08-18

**Authors:** Emmy Verheij, Laura M. Markodimitraki, Robert J. Stokroos, Hans G. X. M. Thomeer

**Affiliations:** ^1^Department of Otorhinolaryngology and Head and Neck Surgery, University Medical Center Utrecht, Utrecht, Netherlands; ^2^Brain Center Rudolf Magnus, University Medical Center Utrecht, Utrecht, Netherlands

**Keywords:** 22q11.2 deletion syndrome, otitis media, mastoidectomy, ossicular chain, otologic surgical procedures

## Abstract

Patients with 22q11.2 deletion syndrome frequently have conductive hearing loss and/or chronic otitis media. Otologic surgery is often opted for. We present two patients undergoing otologic surgery. This case report outlines the typical otologic surgical challenges in patients with 22q11.2 deletion syndrome. Case one is a 52 year old male patient with chronic otitis media who underwent a mastoidectomy. The pre-operative CT scan showed a fused lateral semicircular canal and vestibule. Peroperatively, the lateral semicircular canal could not be used as a landmark to identify the facial nerve. Case two is a 10 year old female patient with conductive hearing loss. A middle ear inspection was performed where a bony epitympanic fixation of the malleus was encountered. In addition, the manubrium of the malleus was atrophic and also fixated. The bony fixation was removed, as was the manubrium of the malleus. Otologists should be aware of these typical anatomical variations in patients with 22q11.2 deletion syndrome. We recommend to use CT scanning of the middle and inner ear when preparing for otologic surgery in 22q11.2 deletion syndrome.

## Introduction

The 22q11.2 deletion syndrome (22q11DS), also known as DiGeorge syndrome or velocardiofacial syndrome, is caused by a microdeletion on chromosome 22 and is the most frequent microdeletion syndrome in humans, occurring in approximately 1 in every 3,000 to 6,000 live births (1). It has a heterogeneous presentation with a broad range of manifestations such as cardiac anomalies, immunodeficiency, velopharyngeal insufficiency and otologic problems. The severity of health issues varies within the 22q11DS patient population (1, 2). Otologic manifestations reported in the literature are conductive or sensorineural hearing loss, the former being the more prevalent. Patients can suffer from recurrent or chronic otitis media, warranting surgical treatment in some cases (3–9). Anatomical malformations of the middle and inner ear have also been described. Among the otologic anatomic malformations found in patients with 22q11DS are ossicular chain anomalies, a malformed lateral semicircular canal and a fused lateral semicircular canal and vestibule (10–12). During mastoidectomy, the lateral semicircular canal is an important landmark for the inner ear in relation to the facial nerve (13). A limited number of reports have been provided thus far regarding 22q11DS and otologic surgery. We report two patients with 22q11DS who underwent otologic surgery and were found to have anatomical malformations of the middle and inner ear. It is of value to be acquainted surgically with the possible anatomical variations, to avoid surgical complications (i.e., deaf ear, iatrogenic damage to the labyrinth or facial nerve) and to plan ossicular chain surgery.

Informed consent was obtained of both described patients and/or parents.

### Case 1

**Figure 1:**
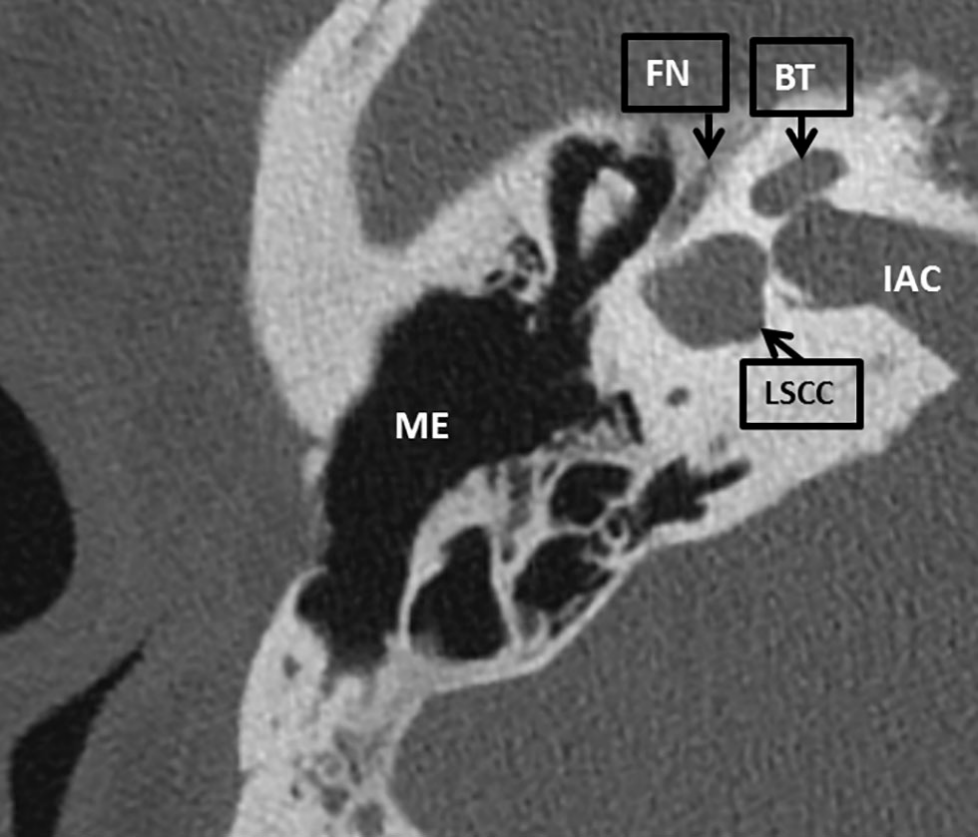
An axial CT scan of the right mastoid bone of patient 1. The lateral semicircular canal is fused with the vestibule to a single cavity. LSCC, lateral semicircular canal; BT, basal turn of the cochlea; IAC, inner auditory canal; FN, facial nerve; ME, middle ear.

A 52 year old man with known 22q11DS has visited our tertiary otologic clinic regularly for 8 years due to chronic middle ear infections, for medical treatment of his chronic otitis media. He had an extensive medical history including diabetes mellitus type 2, morbid obesity, hypertension, Asperger syndrome, asthma, hypoparathyreoidy, anemia, and obstructive sleep apnea. His otorhinolaryngologic history included pharyngoplasty at age four, mastoid and ear surgery including mastoidectomy with attico-antrotomy on the left side at 7 years of age, revision surgeries performed at 11, 27, and 46 years old and chronic rhinosinusitis with nasal polyps. Audiometry tests showed a progression of preexistent mixed hearing loss over time. The CT scan showed a dense stapes superstructure, and the vestibule and lateral semicircular canal were fused to a single cavity (Figure 1) These malformations were present bilaterally. The cochlea was formed normally.

**Figure 2:**
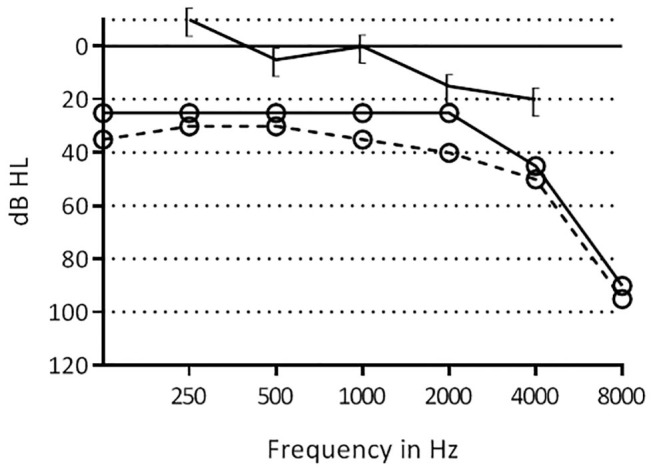
Pre- and post-operative hearing thresholds of the right ear of patient 1. [–[=, postoperative bone conduction thresholds; °-°, postoperative air conduction thresholds, °- -°, preoperative air conduction thresholds.

Due to persistent chronic otitis media he underwent revision surgery on the right side. A meatoplasty was performed to improve the diameter of the introitus of the external auditory meatus. A revision mastoidectomy was performed revealing inflammatory tissue which was removed. The ossicles were intact and freely mobile. Identification of the facial nerve was challenging due to the malformed semicircular lateral canal, the facial nerve taking a relatively more lateral course. No iatrogenic damage to either structure was reported during surgery. The mastoid was obliterated with bone dust, with a bone chip closing the antrum. No cholesteatoma was encountered. Pure tone audiometry 2 months post-surgery was unchanged with a mixed hearing loss bilaterally (Figure 2).

### Case 2

**Figure 3:**
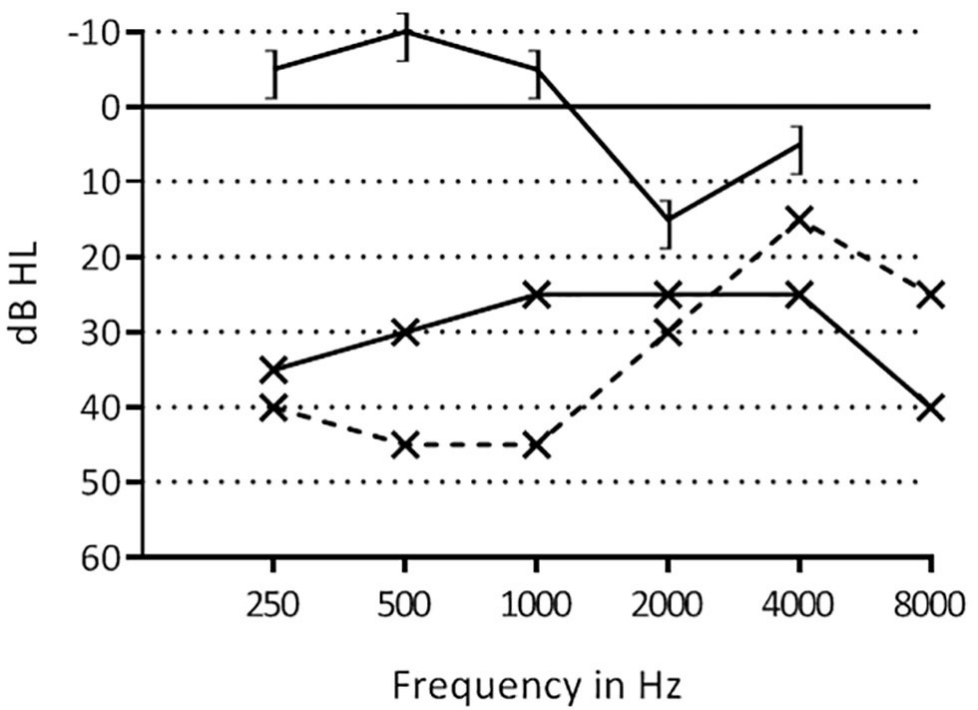
Pre- and postoperative hearing thresholds of patient 2. ] –], postoperative bone conduction thresholds; x–x, postoperative air conduction thresholds; x- -x, preoperative air conduction thresholds.

**Figure 4:**
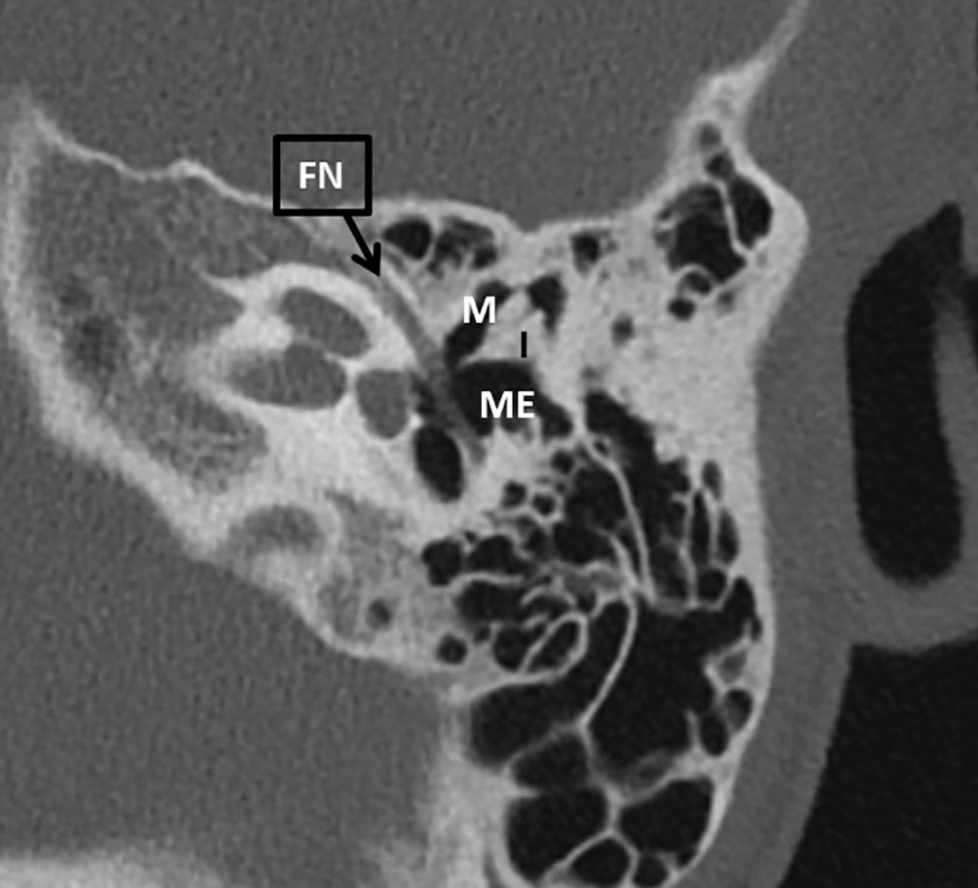
An axial CT scan of the left mastoid bone of patient 2. There is an epitympanic fixation involving the malleus. FN, facial nerve; I, incus; M, malleus; ME, middle ear.

A 10 year old female patient with known 22q11DS was seen at our otorhinolaryngologic department for 8 years. Her relevant medical history included cardiac anomalies (atrial septal defect, ventricular septal defects and pulmonary artery defect), conductive hearing loss bilaterally (Figure 3), malformed ossicular chain and narrow ear canals. The patient underwent tympanostomy tube placement due to Eustachian-tube problems at 3 years of age. The patient wore hearing aids since the age of 4. The indication for middle ear inspection was made, due to a conductive hearing loss and problems wearing hearing aids in combination with glasses. Pre-operatively, a CT scan was performed to assess the middle and inner ear. This showed a more horizontal orientation of the incus, epitympanic ossicular fixation (Figure 4) and dehiscent facial nerve canal on the left side.

A middle ear inspection and ossicular reconstruction on the left side was performed. A retroauricular incision was made, the external auditory canal was saucerized and widened both anteriorly and posteriorly. There was an epitympanic fixation of the malleus, which was curetted. In addition, the manubrium of the malleus was atrophic and anteriorly fixated, and therefore removed. This resulted in a mobile ossicular chain. Audiometry tests showed a hearing improvement of frequencies 0.25, 5, 1, and 2 kHz (Figure 3).

## Discussion

The exact prevalence of clinical otologic manifestations in patients with 22q11DS is unknown in the current literature partly due to reporting heterogeneity (6). Otologists should be aware of the increased risk for middle- and inner ear malformations that have been reported in 22q11DS patients, since typical otologic problems in 22q11DS might need surgical interventions (14). Pre-operative radiologic screening in otologic surgery is warranted in patients with 22q11DS to identify these anatomical malformations.

We present a case of a 22q11DS patient with an abnormally formed lateral semicircular canal, which is an important landmark to identify the facial nerve during surgery in normal temporal bones. This makes identification of the facial nerve more challenging. The nerve took an abnormal, more lateral course in relation the lateral semicircular canal. The facial nerve could be identified using the short process of the incus, and digastric ridge as landmarks (13). In addition, intra-operative facial nerve stimulation is very useful in identifying the facial nerve (15).

Bilateral and unilateral malformations of the lateral semicircular canal are reported to be one of the most common radiological inner ear malformations and are associated with sensorineural as well as conductive hearing loss (16, 17). Inner ear anomalies in 22q11DS patients, concerning the lateral semicircular canal have been reported previously. One case series retrospectively assessing imaging, found a malformed lateral semicircular canal with a small bony island in 33% of the 52 ears, and a lateral semicircular canal and vestibule fused to a single cavity in 29% of ears (11). Another study found a fused vestibule and lateral semicircular canal in 18% of 22 ears, and a wide vestibule in 64% of ears (10). Possibly, a malformed semicircular canal with a small bony island and a wide vestibule describe the same deformity. One case report described a fused lateral semicircular canal and vestibule in one patient and a dysplastic semicircular canal in another patient (12). Another study reported on a patient with poorly formed lateral semicircular canals bilaterally and another patient with bilateral vestibular dilatation (4).

Our second case was a patient with an anomaly of the ossicular chain, resulting in conductive hearing loss. This was a Class III middle ear anomaly, according to the Teunissen Cremers classification (18). A Class III compromises an ossicular chain malformation with a mobile stapes footplate.

Vincent et al. published a case series and literature review analyzing audiometric results following surgical treatment of Teunissen and Cremers Class III patients (19). They reported an postoperative air-bone gap closure tot 10 dB HL or less in 63%, and an postoperative air-bone gap closure to 20 dB or less in 75% (19). In our patient, although her hearing improved mainly in the low frequencies, the air-bone gap postoperatively was 25 dB HL, averaged over frequencies 0.5–4 kHz. In the series by Vincent et al. a malleus fixation was encountered in three patients. In all three cases a bony bridge between the malleus and outer meatus was drilled out, leaving the ossicular chain intact. They had a post-operative air-bone gap of 31 dB HL, 4 dB HL, and 0 dB HL subsequently (19).

Zhan et al. reported on five pediatric cases with an isolated malleus fixation. An ossified mallear ligament was dissected if present, and the bone responsible for the fixation was removed. Postoperatively, they had an air-bone gap of 0–15 dB HL (20). Unlike the cases of Vincent and Zhan et al., in our case the malleus handle was also removed, perhaps explaining the remaining air-bone gap.

In the meta-analysis of Crutcher et al. ossicular chain mobilization was compared to ossiculair chain reconstruction (removing malleus head and incus and reconstructing the ossicular chain) in isolated malleus and/or incus fixation. There was no statistical difference in hearing outcome between the two techniques (21).

In patients with 22q11DS a range of different middle ear malformations are described. Loos et al. described a patient with a malleus with fixation to the tympanic annulus and a thin and horizontally oriented long process of the incus (10). Loos et al. and Verheij et al. both reported patients with 22q11DS with a dense stapes suprastructure (10, 11). In addition, Verheij et al. found a dense manubrium of the malleus (11). A malformation and subluxation of the stapes was described by Cunningham et al. (22). A fusion of the malleus and incus and a monopodal stapes was reported by Devriendt et al. (23). Jiramongkolchai et al. described a bilaterally malformed malleus and incus and a unilateral fusion of the malleus with the lateral wall of the middle ear (4). A recent case report by Kennel et al. described a patient with 22q11DS that underwent middle ear surgery during which an stapes subluxation took place. This unnatural mobility of the stapes was due to an absent stapedial tendon and a weak connection to the oval window (14).

In summary, we present two patients with 22q11DS who underwent otologic surgery. In the first patient the anatomy of the lateral semicircular canal was malformed, challenging the identification of the facial nerve during mastoidectomy surgery. The second patient had an malformed middle ear anatomy. Otologists should be aware of these typical anatomical variations of patients in 22q11DS. We recommend to use CT scanning of the middle and inner ear when preparing for otologic surgery in 22q11.2 deletion syndrome, in addition to intra-operative facial nerve stimulation.

## Data Availability Statement

The raw data supporting the conclusions of this article will be made available by the authors, without undue reservation, to any qualified researcher.

## Ethics Statement

Ethical review and approval was not required for the study on human participants in accordance with the local legislation and institutional requirements. Written informed consent to participate in this study was provided by the participants' legal guardian/next of kin. Written informed consent was obtained from the individual(s), and minor(s)' legal guardian/next of kin, for the publication of any potentially identifiable images or data included in this article.

## Author Contributions

HT and EV initiated the study. LM and EV drafted the manuscript. RS and HT performed the surgery. All authors revised and approved the manuscript.

## Conflict of Interest

The authors declare that the research was conducted in the absence of any commercial or financial relationships that could be construed as a potential conflict of interest.
